# Is urinary oxalate inversely correlated with glomerular filtration rate in chronic kidney disease?

**DOI:** 10.1080/0886022X.2019.1614059

**Published:** 2019-06-04

**Authors:** Cheng Xue, Chenchen Zhou, Jing Xu, Liming Zhang, Shengqiang Yu

**Affiliations:** a Division of Nephrology, Changzheng Hospital, Second Military Medical University, Shanghai, China; b Department of Nephrology, Zhabei Central Hospital of JingAn District of Shanghai, Shanghai, China

Oxalate is a potentially metabolic toxin which is eliminated mainly through the kidney by tubular secretion and glomerular filtration. A recent clinical trial (CRIC study) conducted by Waikar et al. found that higher 24-h urinary oxalate excretion was associated with increased risk of 50% decline in estimated glomerular filtration rate (GFR) and end-stage renal disease (ESRD) in patients with chronic kidney disease (CKD) stages 2–4 [1]. The trial also showed that 24-h urinary oxalate excretion was inversely correlated with eGFR (*r*= −0.13, *p* < .001), which verified the predictive role of urinary oxalate on the risk of CKD progression [1]. However, lower GFR usually leads to unchanged or slightly decreased urinary oxalate excretion when compensatory filtered load increases in early CKD, and leads to decreased urinary oxalate excretion during glomeruli decompensation in advanced CKD. The reduction in eGFR was found to be associated with decreased urine oxalate levels in patients with nephrolithiasis [2]. Few evidence can explain the inverse correlation between urinary oxalate excretion and eGFR in Waikar et al.’s study. In our opinion, body mass index (BMI) and diabetes may partially explain this result. We noticed that patients with urinary oxalate excretion in quintile 5 had higher BMIs and with more diabetes than patients in lower quintiles, and there were statistical differences among different quintiles (*p* < .001) [1]. Higher BMI and diabetes, both associated with higher effective renal plasma flow and glomerular hyperfiltration, could lead to the increased excretion of urinary oxalate [3,4]. Moreover, the associations between oxalate and ESRD became insignificant in subgroups of patients without diabetes and patients with BMI of <32.1, and there was not a dose–response association between urinary oxalate and CKD progression risk [1]. Although multivariable adjustments for BMI and diabetes were performed in the results, there may be sampling bias in the participants of CRIC study, because the BMI of the total sample was relatively high (32.1 ± 7.7) [1]. Besides, protein intake, which impacts renal plasma flow and GFR positively as well, is not available in CRIC study. A recent Turkish study demonstrated a positive correlation of oxalate excretion with increased protein intake and a negative correlation with age in children, which indicated the variation of dietary intake of proteins can affect urinary oxalate excretion [5]. Therefore, the association between 24-h urinary oxalate excretion and eGFR in CKD is complicated and needs more studies to confirm.
